# *Pap1* is an adhesin involved in the interaction of *Sporothrix schenckii* and *Sporothrix brasiliensis* with the host

**DOI:** 10.1016/j.tcsw.2025.100164

**Published:** 2025-12-08

**Authors:** Leonardo Padró-Villegas, Julieta I. Aguilera-Domínguez, Luz A. López-Ramírez, Manuela Gómez-Gaviria, Iván Martínez-Duncker, Laura C. García-Carnero, Joaquín O. Chávez-Santiago, Patricia Ponce-Noyola, Héctor M. Mora-Montes

**Affiliations:** a Departamento de Biología, División de Ciencias Naturales y Exactas, Campus Guanajuato, Universidad de Guanajuato, Noria Alta s/n, col. Noria Alta, C.P., 36050, Guanajuato, Gto., Mexico; b Laboratorio de Glicobiología Humana y Diagnóstico Molecular, Centro de Investigación en Dinámica Celular, Instituto de Investigación en Ciencias Básicas y Aplicadas, Universidad Autónoma del Estado de Morelos, Cuernavaca, Mor. 62209, Mexico

**Keywords:** Cell wall, Adhesin, Biofilm, Immune recognition, Virulence, *Galleria mellonella*, Phagocytosis, Cytokine, Intrinsically disordered protein

## Abstract

*Sporothrix schenckii* and *Sporothrix brasiliensis* are causative agents of sporotrichosis, a mycosis that affects humans and other mammals. Adhesins are considered among the primary virulence factors of these species; however, their molecular identities are currently scarce. Here, we generated silenced mutant strains in *PAP1*, encoding a peptidorhamnomannan-associated protein, in both fungal species and compared their phenotypical characterization. *PAP1*-silenced mutants had normal growth, dimorphism, and morphology, suggesting the gene is not essential. The silenced strains showed defects in adhesion to HeLa cells, laminin, fibronectin, fibrinogen, type-I collagen, and type-II collagen. The *S. schenckii* silenced mutants showed a significant reduction in the adhesion to elastin, whilst the *S. brasiliensis* mutant showed impaired ability to bind thrombospondin 1 and to form biofilms. Both sets of *PAP1*-silenced mutants displayed defects in the cell wall composition, with reduced levels of cell wall rhamnose. The *S. schenckii* mutants showed a compensatory mechanism increasing cell wall mannose content, while the *S. brasiliensis* mutants increased cell wall protein levels in compensation to *PAP1* silencing. Both mutant sets changed their ability to stimulate cytokine production in human peripheral blood mononuclear cells and monocyte-derived macrophages. In addition, phagocytosis of *S. schenckii PAP1*-silenced strains was significantly increased, while the *S. brasiliensis PAP1*-silenced strains were poorly phagocytosed. Finally, *PAP1* silencing affected virulence in both species.

These results suggest that *S. schenckii* and *S. brasiliensis PAP1* code for a protein with adhesive properties to different ligands. This is a cell wall protein that contributes to *Sporothrix* virulence and the interaction with immune cells.

## 1 Introduction

Sporotrichosis is an implantation mycosis that usually affects the skin and subcutaneous tissue (Bonifaz and Vázquez-González, 2013). The most frequent clinical presentation is the lymphangitic form, followed by the fixed cutaneous form, and the disseminated infection, which, although it is a rare form, is associated with high mortality rates (Lopez-Romero et al., 2011; Rojas et al., 2017). The distribution of sporotrichosis is worldwide, and has been reported in all the continents, except Antarctica. However, there are geographical areas with a high burden of sporotrichosis cases, and epidemic outbreaks have been reported in Peru, Mexico, China, Japan, India, South Africa, Madagascar, Australia, Colombia, Brazil, and Venezuela (Chakrabarti et al., 2015; Rasamoelina et al., 2019; Gómez-Gaviria et al., 2023a). The etiological agents belong to the *Sporothrix* genus, and particularly to the pathogenic clade (de Beer et al., 2016). The most frequently isolated species from sporotrichosis cases are *Sporothrix schenckii, Sporothrix brasiliensis,* and *Sporothrix globosa* (Chakrabarti et al., 2015; Lopes-Bezerra et al., 2018a, Hernández-Castro et al., 2022).

This mycosis is not exclusive to human beings; other mammals are also affected, and in veterinary medicine, domestic dogs and cats are the species most affected by *Sporothrix* spp. (Morgado et al., 2022; Santos et al., 2024). Currently, the outbreak of sporotrichosis in Brazil represents the major epidemic focus of this disease worldwide. This is caused by *S. brasiliensis* and has infected thousands of humans and animals (Gremião et al., 2021; Gómez-Gaviria et al., 2023a; Xavier et al., 2023). The infection has spread to neighbouring countries of Brazil and has been reported in Argentina, Paraguay, Panama, Uruguay, and Chile (Gómez-Gaviria et al., 2023a, Xavier et al., 2023). It is noteworthy that *S. brasiliensis* has not been reported outside of South America; the anecdotal cases reported in Europe and the USA were imported from this subcontinent (Gómez-Gaviria et al., 2023a, Xavier et al., 2023). The study of feline sporotrichosis has shown that *S. brasiliensis* can be transmitted by direct contact with yeast-like cells from open skin lesions in cats (Rodrigues et al., 2013), changing the paradigm of the infection route, which was by traumatic contact with plant debris colonized with fungal cells (Lopes-Bezerra et al., 2018a).

*S. schenckii* is a cosmopolitan species, and different from *S. brasiliensis*, it is worldwide distributed and shows a lower virulence profile in experimental models of sporotrichosis (Arrillaga-Moncrieff et al., 2009; Lopes-Bezerra et al., 2018a). Despite both being etiological agents of human and animal sporotrichosis and being associated with different forms of the disease, they have both genetic and phenotypical differences that impact their interaction with the host. The *S. schenckii* genome has over 1200 more genes than the *S. brasiliensis* genome, with shorter intergenic distances, making the *S. schenckii* genome slightly smaller when compared to *S. brasiliensis* (Teixeira et al., 2014). In terms of the proteome, *S. brasiliensis* metabolism is distinct from that of *S. schenckii*, being highly active in amino acid metabolism and cell wall remodeling (Silva-Bailão et al., 2021). The cell wall of both species contains the same components, but the proportions and organization are different (Castro et al., 2013; Martínez-Álvarez et al., 2017; Villalobos-Duno et al., 2021). Consequently, both fungal species are differentially recognized by immune effectors (Batista-Duharte et al., 2018; Kischkel et al., 2022; Galván-Hernández et al., 2023; García-Carnero et al., 2023; Gómez-Gaviria et al., 2023b) and show different virulence levels (Fernandes et al., 2013; Corrêa-Moreira et al., 2021).

Adhesins are among the basic repertoire of virulence factors found in fungal and bacterial pathogens, and they help in the colonization and establishment in host cells and tissues. In *Sporothrix*, adhesins have been mostly studied in *S. schenckii*, perhaps because until 2007 it was considered the sole etiological agent of sporotrichosis (Marimon et al., 2007). *S. schenckii* can adhere to epithelial cells (Sandoval-Bernal et al., 2011) and components of the extracellular matrix (ECM), such as elastin, fibronectin, fibrinogen, laminin, and type-I and type-II collagen (Lima et al., 1999; Lima et al., 2001; García-Carnero et al., 2021; López-Ramírez et al., 2024). In fungi, there are conventional adhesins, which are cell wall proteins transported to the extracellular compartment by the classic secretory pathway, or moonlighting proteins, which have a housekeeping function in the intracellular compartment and, when localized at the cell wall by non-conventional routes, acquire adhesive properties (Cota and Hoyer, 2015; Arvizu-Rubio et al., 2022). So far, in *S. schenckii,* only Gp70, Hsp60, and Pap1 have been described as cell wall adhesins that bind different ECM components and participate in *S. schenckii* virulence (Castro et al., 2013; Rodrigues et al., 2015; García-Carnero et al., 2021; López-Ramírez et al., 2024).

Pap1 is a peptidorhamnomannan-associated protein, intrinsically disordered, and is localized at the cell wall surface, despite lacking a signal peptide. Therefore, its transportation to the extracellular compartment does not follow a classical secretory pathway (García-Carnero et al., 2021). Instead, it is likely the protein enters the endoplasmic reticulum lumen via a signal-peptide-independent mechanism, which recognizes a specific internal sequences within the protein (Rabouille, 2017). Pap1 binds to laminin, elastin, fibrinogen, fibronectin, type-I, and type-II collagens (García-Carnero et al., 2021). Polyclonal anti-Pap1 antibodies blocked the *S. schenckii* ability to kill *Galleria mellonella* larvae, suggesting a role for this protein in virulence (García-Carnero et al., 2021). Besides the observation that *S. brasiliensis* contains a putative ortholog of *S. schenckii PAP1* (García-Carnero et al., 2021), it is currently unknown what the function of its gene product is in this organism.

Here, to assess the biological role of Pap1 in the *S. schenckii* and *S. brasiliensis* interaction with the host, we generated silenced strains in *PAP1* and the phenotype was analyzed, with particular emphasis on the cell wall composition, adhesive properties, interaction with immune cells, and virulence in *G. mellonella*.

## 2 Materials and methods

### 2.1 Culturing conditions

The fungal strains used in this work are listed in Table 1. The parental strains were previously used for genome sequencing (Teixeira et al., 2014) and are considered as reference laboratory strains. The YPD medium, pH 4.5 (1 % [*w*/*v*] yeast extract, 2 % [w/v] gelatin peptone, 3 % [w/v] dextrose, and 2 % [w/v] agarose) was used to grow mycelia and conidia production. For this purpose, cells were incubated in YPD, pH 4.5 plates for 7 days at 28 °C, conidia were harvested, and used to generate biomass in YPD, pH 4.5 broth for 3 days at 28 °C and shaking at 200 rpm. Alternatively, conidia were used to inoculate YPD broth, pH 7.8, and this was incubated for 4 days at 37 °C and 120 rpm. Under these conditions, more than 96.2 ± 2.3 % cells were yeast-like cells, as reported (Martínez-Álvarez et al., 2017). Yeast-like cells were harvested by centrifuging at 4 °C, washed three times with deionized water, and immediately used in assays described below. When required, cells were inactivated at 60 °C for 2 h, as described (Martínez-Álvarez et al., 2017). Fungal transformation was performed with *Agrobacterium tumefaciens* AGL-1. This was grown in LB medium (0.5 [w/v] yeast extract, 1 % [w/v] gelatin peptone, and 1 % [w/v] NaCl) at 28 °C. Upon transformation, fungal cells were selected on YPD plates supplemented with 25 μg mL^−1^ nourseothricin (GoldBio, St Louis, MO, USA), pH 4.5 (Tamez-Castrellón et al., 2018).

**Table 1:** *Sporothrix schenckii* and *Sporothrix brasiliensis* strains used in this study.

Strain	Genotype	*PAP1* silencing (%)^a^	pCambia-Nou copy number^b^	Reference
*S. schenckii*				
1099–18 ATCC MYA 4821	Wild-type	0.0 ± 0.0	N.D.	(Castro et al., 2013)
HSS61	1099–18 ATCC MYA 4821 transformed with pCambia-Nou-PAP1	96.7 ± 3.2	1.2 ± 0.3	This work
HSS62	1099–18 ATCC MYA 4821 transformed with pCambia-Nou-PAP1	98.7 ± 2.9	1.1 ± 0.2	This work
HSS63	1099–18 ATCC MYA 4821 transformed with pCambia-Nou-PAP1	98.9 ± 3.5	0.9 ± 0.2	This work
HSS64	1099–18 ATCC MYA 4821 transformed with pCambia-Nou-PAP1	97.3 ± 2.8	1.4 ± 0.1	This work
HSS65	1099–18 ATCC MYA 4821 transformed with pCambia-Nou-PAP1	95.8 ± 2.7	1.2 ± 0.2	This work
HSS67	1099–18 ATCC MYA 4821 transformed with pCambia-Nou	1.6 ± 1.2	1.3 ± 0.4	(Gómez-Gaviria et al., 2025)
HSS68	1099–18 ATCC MYA 4821 transformed with pCambia-Nou	1.4 ± 0.9	0.9 ± 0.3	(Gómez-Gaviria et al., 2025)
*S. brasiliensis*				
5110 ATCC MYA 4823	Wild-type	0.0 ± 0.0	N.D.	(Castro et al., 2013)
HSB30	5110 ATCC MYA 4823 transformed with pCambia-Nou-PAP1	98.2 ± 1.8	1.2 ± 0.4	This work
HSB31	5110 ATCC MYA 4823 transformed with pCambia-Nou-PAP1	98.6 ± 0.9	1.4 ± 0.2	This work
HSB32	5110 ATCC MYA 4823 transformed with pCambia-Nou-PAP1	95.7 ± 3.2	1.1 ± 0.4	This work
HSB33	5110 ATCC MYA 4823 transformed with pCambia-Nou-PAP1	97.7 ± 3.8	1.2 ± 0.1	This work
HSB34	5110 ATCC MYA 4823 transformed with pCambia-Nou-PAP1	95.1 ± 3.3	1.4 ± 0.5	This work
HSB28	5110 ATCC MYA 4823 transformed with pCambia-Nou	1.2 ± 0.5	1.1 ± 0.2	This work
HSB29	5110 ATCC MYA 4823 transformed with pCambia-Nou	1.5 ± 0.6	1.3 ± 0.2	This work

Castro, R. A., P. H. Kubitschek-Barreira, P. A. C. Teixeira, G. F. Sanches, M. M. Teixeira, L. P. Quintella, S. R. Almeida, R. O. Costa, Z. P. Camargo, M. S. S. Felipe, W. de Souza and L. M. Lopes-Bezerra (2013). “Differences in cell morphometry, cell wall topography and Gp70 expression correlate with the virulence of *Sporothrix brasiliensis* clinical isolates.” PLoS ONE**8**(10): e75656.

Gómez-Gaviria, M., J. A. Martínez-Álvarez, I. Martínez-Duncker, A. R. S. Baptista and H. M. Mora-Montes (2025). “Silencing of *MNT1* and *PMT2* shows the importance of *O*-linked glycosylation during the *Sporothrix schenckii*-host interaction.” J Fungi (Basel)**11**(5): 352.

a Analyzed by RT-qPCR. ^b^ Analyzed by qPCR. In both essays, the gene encoding the ribosomal protein L6 was used for data normalization. Data are means ± SD of three independent experiments performed in duplicat.

### 2.2 Generation of mutant strains

We used the gene silencing strategy as this has been successfully used in both *S. schenckii* and *S. brasiliensis* (Lozoya-Pérez et al., 2018; Padró-Villegas et al., 2025). The binary plasmid pCambia-Nou was used, which confers resistance to nourseothricin (Tamez-Castrellón et al., 2018). A 212 bp fragment was amplified from *S. schenckii* genomic DNA using the primer pair 5’-CTCGAGTCCATCGTCAACAAGATCAAGGA and 5’-AAGCTTGAATCATCGCCGTAGCCG (underlined sequences represent added *Xho*I and *Hin*dIII sites for cloning), and the amplicon was cloned into the XhoI and HindIII sites of pCambia-Nou, generating pCambia-Nou-Sense. The same fragment was amplified in antisense with the primer pair 5’-AGGCCTGAATCATCGCCGTAGCCG and 5’-GGGCCCTCCATCGTCAACAAGATCAAGGA (underlined sequences are added *Stu*I and *Apa*I sites for cloning), and used to clone into the StuI and ApaI sites of pCambia-Nou-Sense, generating pCAmbia-Nou-PAP1. The 212 bp *PAP1* fragment spans from position 7 to 219 of the open reading frame and is 100 % identical in both *S. schenckii* and *S. brasiliensis*. This construction was used to transform *A. tumefaciens* AGL-1, and these cells were used to perform *Agrobacterium tumefaciens*-mediated transformation as reported (Lozoya-Pérez et al., 2018). Transformants growing in the presence of 25 μg mL^−1^ nourseothricin were selected through five monoconidial passages, and three dimorphism rounds in YPD, pH 7.8 (Lozoya-Pérez et al., 2018).

### 2.3 Analysis of gene silencing and quantification of pCambia-Nou insertional events

Total RNA was extracted from yeast-like cells as described (Robledo-Ortiz et al., 2012), cDNA was synthesized and purified by chromatography (Trujillo-Esquivel et al., 2016; Trujillo-Esquivel et al., 2017), and quantified in a NanoDrop 2000 (Thermo Fisher Scientific, Waltham, MA, USA). RT-qPCR was performed in a thermocycler StepOne Plus (Life Technologies, Carlsbad, CA, USA), and data were analyzed with the StepOne software V 2.2 (Life Technologies) and the 2^-∆∆Ct^ method (Livak and Schmittgen, 2001). The reactions contained SYBR Green PCR Master Mix (Life Technologies) and the primer pair 5’-TCCATCGTCAACAAGATCAAGGA and 5’-GAATCATCGCCGTAGCCG that amplifies the 212 bp fragment cloned into the pCambia-Nou vector. For data normalization, the gene encoding the ribosomal protein L6 was amplified with the primers 5’-ATTGCGACATCAGAGAAGG and 5’-TCGACCTTCTTGATGTTGG, as this was previously reported to be a constitutive expression gene in *S. schenckii* and *S. brasileinsis* (Trujillo-Esquivel et al., 2017; Padró-Villegas et al., 2025). For gene expression analysis under different conditions, RNA was extracted from cells incubated as described in the following sections. To quantify the number of pCambia-Nou insertional events, we used genomic DNA and qPCR using the primer pair 5′-TAAGAGAGGTCCGCAAGTAGATT and 5′-TTAGGGGGGCAGGCAGGGCATGC, which amplifies a fragment of the gene conferring resistance to nourseothricin (Gómez-Gaviria et al., 2025).

### 2.4 Bioinformatics analysis

The predicted Pap1 three-dimensional model from *S. schenckii* and *S. brasiliensis* was retrieved from the UniProt database (accessed on Sep 3rd, 2025) (Consortium, 2022). The files were then analyzed with PyMOL software (version 3.0) (https://www.pymol.org/) to visualize protein three-dimensional structures. The protein structures were analyzed using CB-Dock (version 2.0) (Liu et al., 2020) to predict putative binding cavities involved in interactions with other proteins. Docking analyses were performed with the HDOCK server (version 1.1) (Yan et al., 2020), using the Pap1 strutures and the following putative ligands: thrombospondin-1 (UniProtKB: 1LSL), laminin (UniProtKB: 4YEQ), elastin (UniProtKB: P15502), fibrinogen (UniProtKB: 3GHG), fibronectin III (UniProtKB: 1FNF), type-I collagen (UniProtKB: 8K4W), type-II collagen (UniProtKB: 9J1R), and bovine serum albumin (BSA; UniProtKB: 4F5S). PyMOL was used to visualize the protein–protein complexes and to identify interacting residues within the predicted binding sites.

### 2.5 Cell adhesion assays

The adhesion to ECM was analyzed by ELISA, as reported (Lima et al., 1999). Nunc MaxiSorp™ flat-bottom 96-well microplates were incubated with 0.05 % (*w*/*v*) PBS-Tween 20 and 1 μg of the ECM component for 3 h at room temperature. The coating proteins were: human laminin, human elastin, human fibrinogen, human fibronectin, human thrombospondin-1, human type-I collagen, or bovine type-II collagen (all from Sigma-Aldrich). Plates were incubated overnight with 1 % (*w*/*v*) BSA in PBS at 4 °C. Then, aliquots of 100 μL, containing 5 × 10^6^ yeast-like cells, were added per well, and plates were incubated for 1 h at 37 °C. Plates were washed with PBS-0.05 % (*v*/v) Tween 20 (PBS-Tween), and 100 μL of rabbit polyclonal anti-rHsp60 diluted at 1:3000 was added (García-Carnero et al., 2021). Plates were incubated for 2 h at room temperature, washed with PBS-Tween, and 100 μL of goat anti-rabbit IgG-peroxidase antibody diluted 1:5000 (Sigma-Aldrich) was added, and incubated for 2 h at room temperature. Plates were washed with PBS-Tween, and 0.1 mg mL^−1^ 2,2′-azino-bis(3-ethylbenzothiazoline-6-sulfonic acid) diammonium salt and 0.006 % (v/v) hydrogen peroxide were added to wells and incubated at room temperature for 20 min. Colour development was stopped with 2 N sulfuric acid, and absorbance was read at 450 nm in a Varioskan LUX Multimode Microplate Reader (Thermo Fisher Scientific).

In some experiments, HeLa cells (ATCC) were grown in monolayers in Eagle's Minimum Essential Medium (EMEM, Sigma-Aldrich) at 37 °C and 5 % CO_2_ (v/v). Then, monolayers were incubated with 0.25 % (w/v) trypsin and 0.53 mM EDTA (Sigma-Aldrich) (García-Carnero et al., 2021), cells were washed twice with chilled PBS, cell concentration adjusted at 5 × 10^6^ cells mL^−1^, and 200 μL were seeded in 96-well microplates. Plates were incubated for 24 h at 37 °C and 5 % (v/v) CO_2_, blocked with BSA as previously described, and used in adhesion assays.

### 2.6 Biofilm formation assays

Aliquots of 100 μL of 1 × 10^7^ cells mL^−1^ were placed in flat-bottom Nunc polystyrene 96-microtiter plates (Thermo Fisher Scientific). Plates were incubated for 4 h at 37 °C, washed three times with PBS to remove non-adherent cells, 100 μL RPMI-1640 medium supplemented with l-glutamine (Sigma-Aldrich) was added per well, and further incubated for 24 h at 37 °C. Plates were washed five times with PBS, 100 μL of absolute methanol was added, incubated for 15 min at room temperature, the methanol was removed, and the plates were air-dried. Then, 100 μL of 0.02 % (*w*/*v*) crystal violet was added to each well, incubated for 20 min at room temperature, washed three times with deionized water, 150 μL of 33 % (v/v) acetic acid was added per well, and the absorbance at 590 nm was measured (Clavijo-Giraldo et al., 2023).

The biofilm extracellular matrix was isolated as reported (Al-Fattani and Douglas, 2006). Briefly, the RPMI-1640 medium was removed, 200 μL of 50 μg mL^−1^ chitinase from *Streptomyces griseus* (Sigma-Aldrich) was added, and plates were incubated at 25 °C for 2 h. Wells were sonicated for 5 min with a Q700 Sonicator (Thomas Scientific, Swedesboro, NJ, USA) and centrifuged to pellet cells. The supernatant was saved, passed through an Amicon Ultra centrifugal filter with Ultracel-3 K (Sigma-Aldrich), and used to detect glucose and glucosamine by High-Performance Anion-Exchange Chromatography with Pulsed Amperometric Detection (HPAEC-PAD) in a Dionex system (Thermo Fisher Scientific), as reported (Plaine et al., 2008). Total protein content was quantified with the Pierce BCA Protein Assay (Thermo Fisher Scientific). The pelleted cells were resuspended in 500 μL, the tubes were incubated for 30 min in static conditions to allow sedimentation of cell aggregates, and the amount of free cells in the supernatant was quantified with a hemocytometer.

### 2.7 Analysis of cell wall composition

Yeast-like cells were grown for 4 days at 37 °C in YPD, pH 7.8, harvested by centrifuging, and disrupted in an MSK cell homogenizer (Braun, Melsungen, Germany), with at least six disruption rounds of 1 min and resting in an ice bed in between (Mora-Montes et al., 2010). The homogenates were centrifuged at 10,000 x*g* for 10 min at 4 °C. The pellet was saved and serially incubated with hot SDS, β-mercaptoethanol, and NaCl, with deionized water washing between treatments (Mora-Montes et al., 2007). The cleansed cell walls were pelleted by centrifuging, freeze-dried, and hydrolyzed with 2 M trifluoroacetic acid (Sigma-Aldrich), as described (Mora-Montes et al., 2007). Samples were analyzed by HPAEC-PAD in a Dionex system (Thermo Fisher Scientific). Samples were separated in a CarboPac PA-1 column with a pre-guard CarboPac PA-1 column, using 3.5 % (w/v) 200 mM NaOH in an isocratic gradient with a flux rate of 1 mL min^−1^. Absolute values were obtained after monosaccharide content was colorimetrically quantified in one mg of dry cell wall (Dubois et al., 1956).

For analysis of the cell wall *O*-linked or *N*-linked glycans content, yeast-like cells were adjusted to 1 × 10^9^ cells, and these were either β-eliminated (Diaz-Jimenez et al., 2012) or incubated for 20 h at 37 °C with 25 U endoglycosidase H (New England Biolabs), to release *O*-linked glycans and *N*-linked glycans, respectively. In both treatments, cells were centrifuged, glycans were saved from the supernatants and used for total sugar quantification by a colorimetric method (Dubois et al., 1956) and for acid hydrolysis followed by HPAEC-PAD, as described above. Cell wall protein quantification was performed after walls were alkali hydrolyzed (Mora-Montes et al., 2007) and using the Pierce BCA Protein Assay.

### 2.8 Ethics statement

The study was approved by the Institutional Research Ethics Committee of Universidad de Guanajuato (Ref. CEPIUG-P13–2023). The use of primary human cells was performed in accordance with the Declaration of Helsinki. Venous blood samples were withdrawn from adult and healthy volunteers who signed an informed consent.

### 2.9 Cytokine stimulation in human peripheral blood mononuclear cells and monocyte-derived macrophages

Human EDTA-treated venous blood was mixed with Histopaque-1077 (Sigma-Aldrich), and PBMCs were separated by differential centrifugation, as described (Endres et al., 1988). Human-fungal cell interactions were performed in round-bottom 96-well microplates. Cell concentration of both human and fungal cells was adjusted using RPMI 1640 Dutch modification (supplemented with 2 mM glutamine, 0.1 mM pyruvate, and 0.05 mg mL^−1^ gentamycin; all reagents from Sigma-Aldrich). Each well contained 100 μL 1 × 10^5^ yeast-like cells and 100 μL 5 × 10^5^ PBMCs, and plates were incubated for 24 h at 37 °C with 5 % (*v*/v) CO_2_. Plates were centrifuged for 10 min at 3000 x*g* at 4 °C, and the supernatants were used for cytokine quantification. All plates included mock wells, where only human PBMCs were included. Cytokine quantification from these wells was considered the threshold levels that were subtracted from the experimental wells.

When required, the human cells were pre-incubated for 1 h at 37 °C and 5 % (v/v) CO_2_ with one of the following immune receptor antagonists: 10 μg mL^−1^ of anti-mannose receptor (MR) (Thermo-Fisher Scientific, MA5–44033), or 10 μg mL^−1^ anti-TLR4 antibody (Santa Cruz Biotechnology, Dallas, TX, USA sc-293,072). As control, 10 μg mL^−1^ irrelevant IgG1 antibody (Santa Cruz Biotechnology, Cat. No. sc-52,003), same isotype antibody as the anti-MR and anti-TLR4, was used in preincubations with the human cells. All the tested antibodies were negative in the *Limulus* amebocyte lysate reaction to detect bacterial lipopolysaccharide. However, all the interactions where preincubated cells were included were performed in the presence of 5 μg mL^−1^ polymyxin B (Sigma-Aldrich) as described (Martínez-Álvarez et al., 2017).

Monocyte-derived macrophages were obtained by stimulating human PBMCs with recombinant human granulocyte-macrophage colony-stimulating factor (Sigma-Aldrich), as reported (Perez-Garcia et al., 2016). These monocyte-derived macrophages were used like human PBMCs to stimulate cytokine production and in preincubation steps with an immune receptor antagonist.

In both cases, interaction with PBMCS and monocyte-derived macrophages, cytokine levels were measured with DuoSet ELISA Development kits (R&D Systems, Minneapolis, MN, USA). The analyzed cytokines were interleukin-1β (IL-1β), interleukin-6 (IL-6), interleukin-10 (IL-10), and tumor necrosis factor-alpha (TNFα).

### 2.10 Analysis of phagocytosis

Fungal cell phagocytosis was analyzed by flow cytometry in a FACSCanto II cytometer connected to a FACSDiva acquisition system (Becton Dickinson, Franklin Lakes, NJ, USA), as previously reported (González-Hernández et al., 2017; Gómez-Gaviria et al., 2023b). Fifty thousand events were collected per biological replicate, gating for immune cells, previously stained with 1.25 mg mL^−1^ Trypan Blue. Since fungal cells were labeled with Acridine Orange, once within the mature phagolysosome were detected in the red fluorescence channel, and were considered in the late stage of phagocytosis (Abrams et al., 1983). In some experiments, monocyte-derived macrophages were preincubated with antagonistic antibodies against immune receptors, as described in section 2.9.

### 2.11 *Galleria mellonella* infection

Fungal virulence was analyzed in *Galleria mellonella* larvae, where an alternative model of disseminated sporotrichosis has been previously established (Clavijo-Giraldo et al., 2016). Larvae were from a previously established colony in the laboratory. Larvae with a body length larger than 1.0 cm and a typical creamy colour were included in the study. Fungal inoculation was performed in the last left pro-leg with 10 μL of a suspension at 1 × 10^7^ yeast-like cells mL^−1^, as described (Clavijo-Giraldo et al., 2016). Groups containing 30 larvae were used for each fungal strain. To control the mechanical stress of inoculation, a control group injected with PBS was included. Larvae mortality was recorded daily for two weeks. Dead larvae or those alive at the end of the observation period were decapitated, hemolymph withdrawn and anticoagulated, and used for colony-forming units quantification, as described (García-Carnero et al., 2020).

To analyze immunological parameters, named hemocyte levels and phenol oxidase activity, 10 larvae per fungal strain were inoculated as mentioned, and hemolymph was withdrawn at 24 h post-inoculation. Hemocytes were quantified in a hemocytometer, whilst phenol oxidase activity was measured as described (Lozoya-Pérez et al., 2020). Briefly, reactions were carried out in 96-well microplates, containing 100 μg protein from cell-free hemolymph and 20 mM 3,4-dihydroxyDL-phenylalanine (Sigma-Aldrich). The initial absorbance at 490 nm was measured, and reactions were incubated for 30 min at 37 °C, followed by a second measurement of absorbance at 490 nm. Enzyme activity was defined as the change in the absorbance at 490 nm min^−1^ μg protein^−1^ (Lozoya-Pérez et al., 2020). This hemolymph was also used to measure a well-known marker of cell cytotoxicity, cell-free lactate dehydrogenase (Shekhawat et al., 2025), with the Pierce LDH Cytotoxicity Assay (Thermo Fisher Scientific).

### 2.12 Statistical analysis

Results were analyzed with the GraphPad Prism 8 software. Results were initially analyzed with Dunnett's test. For the data with a *P* value <0.05, normality was analyzed with the Shapiro-Wilk test. Results showing normality were analyzed with the unpaired *t*-test. Immunological parameters generated with both human and insect immune cells were analyzed with Mann-Whitney *U* tests, since they did not show normality. A total of eight healthy human donors were included in the study, and samples were assayed in duplicates. Larva survival experiments were analyzed with the Log-rank test, and each fungal strain was analyzed in groups containing 30 larvae. In all cases, statistical significance was set at *P* < 0.05.

## 3 Results

### 3.1 Generation of *PAP1*-silenced mutants in *Sporothrix schenckii* and *Sporothrix brasiliensis*

The *S. schenckii PAP1* (GenBank accession code SPSK_00848) has a putative functional ortholog in *S. brasiliensis* (GenBank accession code SPBR_07403), which is referred here as *S. brasiliensis PAP1*. We used the previously designed pCambia-Nou plasmid (Tamez-Castrellón et al., 2018) as the backbone to construct a binary vector with sense and anti-sense regions of the *PAP1* open reading frame. We selected the beginning of the coding region because this is 100 % identical in both species. Following the *A. tumefaciens*-mediated transformation, we obtained 378 and 423 transformants of *S. schenckii* and *S. brasiliensis*, respectively, and after the enrichment of transformant nuclei with monoconidial passages and dimorphism stimulation, 102 and 135 transformants of *S. schenckii* and *S. brasiliensis*, respectively, we selected for molecular analyses. First, we confirmed the presence of the pCambia-Nou binary plasmid by PCR, amplifying the gene that confers resistance to nourseothricin (data not shown). Next, we analyzed the *PAP1* expression levels by RT-qPCR, and the first five strains with high levels of gene silencing were selected for each species. All the strains used in the following experiments showed a *PAP1* silencing higher than 95 % (Table 1). For *S. schenckii*, strains selected were HSS61-HSS65, and for *S. brasiliensis* were HSB30-HSB34 (Table 1). All of them showed a single insertional event of the binary plasmid within the genome (Table 1). It is worth noting that our strategy does not control the binary plasmid insertional point; therefore, polar effects may be observed in the mutant cells. For this reason, we analyzed five strains per species. The consistency of phenotypes across mutant strains will minimize the idea of polar effects because of the molecular strategy. In addition, to analyze the contribution of the binary vector to the phenotype, the wild-type (WT) strains were also transformed with the empty pCambia-Nou. This generated strains HSS67 and HSS68 in *S. schenckii,* and HSB28 and HSB29 in *S. brasiliensis,* which had only one copy of the binary plasmid within the genome (Table 1).

There were no changes in colony or cell morphology in the mutant strains when compared with the WT strains, nor were there defects in the doubling times, which were 3.6 ± 0.9 h for filament cells and 8.5 ± 0.9 h for yeast-like cells, on average for mutant cells of both species. WT strains showed doubling times of 3.2 ± 0.5 h for filament cells and 8.2 ± 0.6 h for yeast-like cells.

**Fig. 1:**
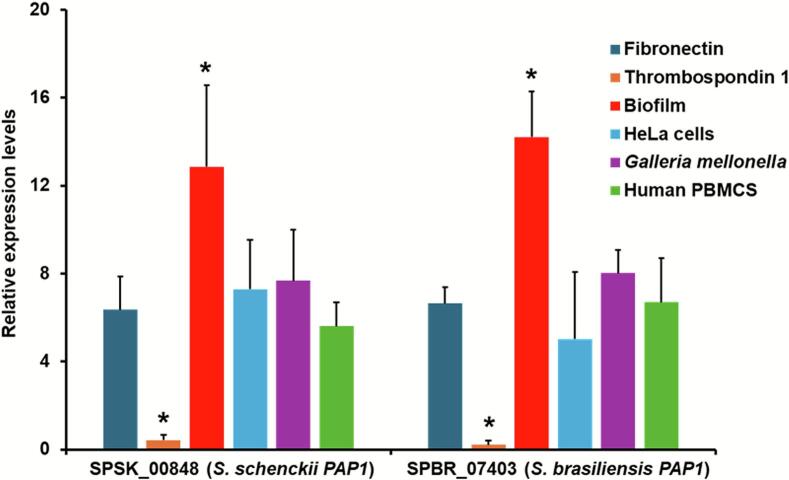
***PAP1* expression under different growing conditions*.*** Yeast-like cells were incubated under the following conditions at 37 °C: 1 h with either 1.0 μg mL^−1^ fibronection or thrombospondin 1; 1 h with a monolayer of HeLa cells; 1 h with 5 × 10^6^ human PBMCs, or injected in the hemolymph of *Galleria mellonella* larvae and incubated for 24 h. Alternatively, biofilms were matured for 48 h at 37 °C. From these conditions, total RNA was extracted, cDNA synthesized with oligo(dT) primer (20 mer), and *PAP1* expression quantified by RT-qPCR. Data were normalized using the expression of the gene encoding the ribosomal protein L6 and yeast-like cells growth in YPD at 37 °C as reference conditions (point zero on the Y axis). Results are means ± SD of three independent experiments performed in duplicate. The Dunnett's test and then the unpaired *t*-test were used for data analysis. *P < 0.05 when compared to the other growing conditions.

Previous results indicated that *S. schenckii PAP1* is expressed about 3-fold higher in yeast-like cells than in micelium (Giosa et al., 2020). Here, we analyzed the expression in yeast-like cells and filament cells again and found that *PAP1* is highly expressed in yeast-like cells of both *S. schenckii* and *S. brasiliensis* (3.7 ± 1.4 fold and 4.3 ± 1.1 fold, respectively). Next, we analyzed the expression of yeast-like cells growing under different conditions, and we took the *PAP1* expression in YPD-grown yeast-like cells as the reference condition (point zero in the Y axis of Fig. 1). The *PAP1* expression in both species showed a similar trend, upregulation in yeast-like cells incubated with fibronectin, HeLa cells, or human PBMCs, cells growing in biofilms, and cells growing in the hemolymph of *G. mellonella* larvae (Fig. 1). The highest expression was observed in cells growing in biofilms (Fig. 1). Previous results indicated that *S. schenckii* Pap1 does not interact with thrombospondin 1 (García-Carnero et al., 2021). Thus, it was feasible to hypothesize that cells interacting with this extracellular matrix component may not have a significant upregulation of *PAP1*. Results in Fig. 1 confirmed this hypothesis and suggest that *PAP1* upregulation responds to particular stimuli.

### 3.2 *PAP1* silencing affected the *Sporothrix schenckii* and *Sporothrix brasiliensis adhesive properties*

*S. brasiliensis* Pap1 has an 79 % identity with *S. schenckii* Pap1, containing 360 amino acids, 55 more than the *S. schenckii* protein. This generates four gaps when both protein sequences are aligned, with a 41 amino acid gap as the biggest, spanning from amino acid 180 to 220 of *S. brasiliensis* Pap1. Because of these differences, we performed a bioinformatics analysis to predict putative adhesive properties of *S. brasiliensis* Pap1. Similar to *S. schenckii* Pap1, the *S. brasiliensis* is predicted to lack a signal peptide and to be classified as a highly disordered protein (analyzed at https://services.healthtech.dtu.dk/cgi-bin/webface2.cgi?jobid=68B9AD7A00010DD3BC9BACE7&wait=20 and https://iupred2a.elte.hu/, respectively). The putative three-dimensional structure of both proteins was retrieved from the UniProt database and used to perform in silico docking experiments, using different proteins from the ECM, which have been demonstrated previously as ligands of *S. schenckii* Pap1 (García-Carnero et al., 2021). The in silico results generated with *S. schenckii* Pap1 fitted well the experimental evidence for this protein (García-Carnero et al., 2021): high probability of binding to laminin, elastin, fibrinogen, fibronectin, and type-I and type-II collagen (Table 2). As previously demonstrated (García-Carnero et al., 2021), the protein is unlikely to bind to thrombospondin 1 or BSA. For *S. brasiliensis* Pap1, the in silico analysis did not predict interaction with BSA and possible interaction with elastin (Table 2). For thrombospondin 1, laminin, fibrinogen, fibronectin, and type-I and type-II collagen, the protein showed a high probability of binding (Table 2).

**Table 2:** In silico docking analysis between ***Sporothrix schenckii*** and *Sporothrix brasiliensis* Pap1 with selected extracellular matrix proteins.

**Protein**	**Ligand**	**Docking Score**⁎	**Confidence Score**⁎⁎
*Sporothrix schenckii* Pap1	Thrombospondin 1	−163.83	0.4061
	Bovine serum albumin	−153.46	0.4613
	Laminin	−223.36	0.8042
	Elastin	−212.58	0.8389
	Fibrinogen	−261.33	0.8573
	Fibronectin	−277.48	0.8594
	Collagen type I	−223.29	0.8124
	Collagen type II	−219.96	0.8021
*Sporothrix brasiliensis* Pap1	Thrombospondin 1	−200.2	0.7318
	Bovine serum albumin	−159.04	0.3859
	Laminin	−208.51	0.8632
	Elastin	−185.94	0.6148
	Fibrinogen	−256.17	0.8932
	Fibronectin	−276.62	0.7912
	Collagen type I	−225.72	0.8197
	Collagen type II	−217.27	0.7934

⁎ Docking scores were used as a comparative metric between different docking complexes, and a more negative score indicated a more favorable binding model (Yan et al., 2020).

⁎⁎ A confidence score over 0.7 suggested a high likelihood of molecular interaction; a score from 0.5 to 0.7 indicated the interaction is likely; and scores below 0.5 were associated with no binding between proteins (Yan et al., 2020).

**Fig. 2:**
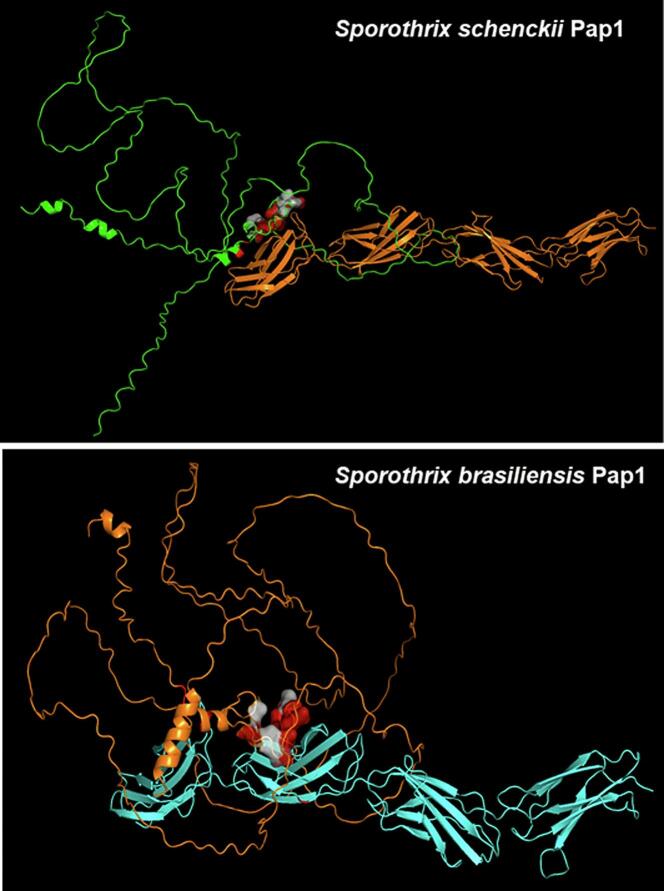
**In silico docking between Pap1 and fibronectin.** In the upper panel, *Sporothrix schenckii* Pap1 is in green, and fibronectin is in orange. In the lower panel, *Sporothrix brasiliensis* Pap1 is in orange and fibronectin is in blue. In both panels, the surface pocket involved in binding is in grey, while the amino acids interacting with the ligand are in red. Both images were generated with PyMol. (For interpretation of the references to colour in this figure legend, the reader is referred to the web version of this article.)

The analysis also allowed the prediction of a putative surface pocket involved in the interaction with the ligands in both proteins, and this was the same in both cases, involving amino acids ^248^SGGNFSAGATR^258^ and ^304^SGGNFSAGATR^314^ for *S. schenckii* Pap1 and *S. brasiliensis* Pap1, respectively. The putative surface pocket with binding properties is shown in Fig. 2.

**Fig. 3:**
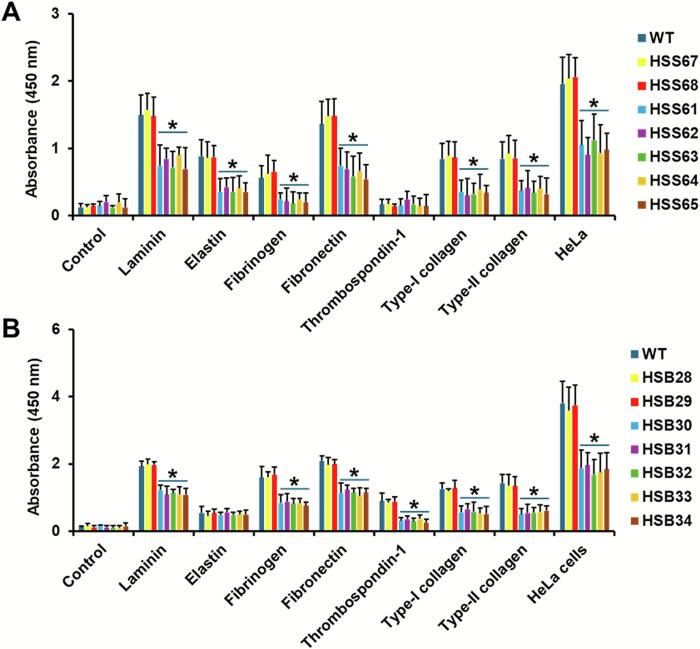
**Adhesion to extracellular matrix components and HeLa cells of *Sporothrix schenckii* and *Sporothrix brasiliensis PAP1*-silenced strains.** The indicated extracellular matrix component was used to coat 96-well plates, then yeast-like cells were added, unbound cells were removed by extensive washing, and adherent cells were detected by ELISA with a primary anti-rHsp60 antibody. Control refers to wells coated with bovine serum albumin. Panel **A** contains data generated with *S. schenckii,* and WT is the 1099–18 ATCC MYA 4821 strain. Panel **B** shows results with *S. brasiliensis* cells, and the WT is the 5110 ATCC MYA 4823 strain. Alternatively, for both panels, 1 × 10^6^ HeLa cells were placed per well, incubated 24 h at 37 °C and 5 % (*v*/v) CO_2_, and used in the adhesion assays. Results are means ± SD of three biological replicates performed in duplicate. The Dunnett's test and then the unpaired t-test were used for data analysis. In both panels, * *P* < 0.05 when compared to WT or strains HSB1 and HSB2.

Next, we analyzed the ability of the silenced mutants to adhere to ECM components. For *S. schenckii*, the WT and control strains HSS67 and HSS68 showed similar binding abilities, with high binding to laminin and fibrinogen, intermediate binding to elastin, fibrinogen, and type-I and type-II collagen (Fig. 3A). The *PAP1*-silenced strains (HSS61-HSS65) showed a significant reduction in the ability to bind these ECM components (Fig. 3A). No binding to thrombospondin 1 or BSA (control well) was observed for all the analyzed strains (Fig. 3A). For *S. brasiliensis*, the WT and control strains (HSB28 and HSB29) also showed a similar profile of binding: high binding to laminin, fibrinogen, and fibronectin, moderate binding to thrombospondin 1, type-I and type-II collagen, and low binding to elastin (Fig. 3B). The *PAP1*-silenced mutants (HSB30-HSB34) showed reduced ability to bind the tested ECM components, except elastin, where no significant differences were observed when compared to the WT strain (Fig. 3B). Threshold binding was observed in the control wells, where only BSA was used to block the plastic surface (Fig. 3B).

We also assessed the ability to bind human epithelial cells, and for this purpose, we used the HeLa cell line. Both species were capable of binding the cell line, but *S. brasiliensis* cells bound to the cell line in a higher proportion than *S. schenckii* cells (see Y axes in Fig. 3A and B; *p* < 0.05). In both species, the control strains bound to the cell line as the WT strains, and a significant reduction in the binding of the *PAP1*-silenced mutant to HeLa cells was observed (Fig. 3A and B).

**Fig. 4:**
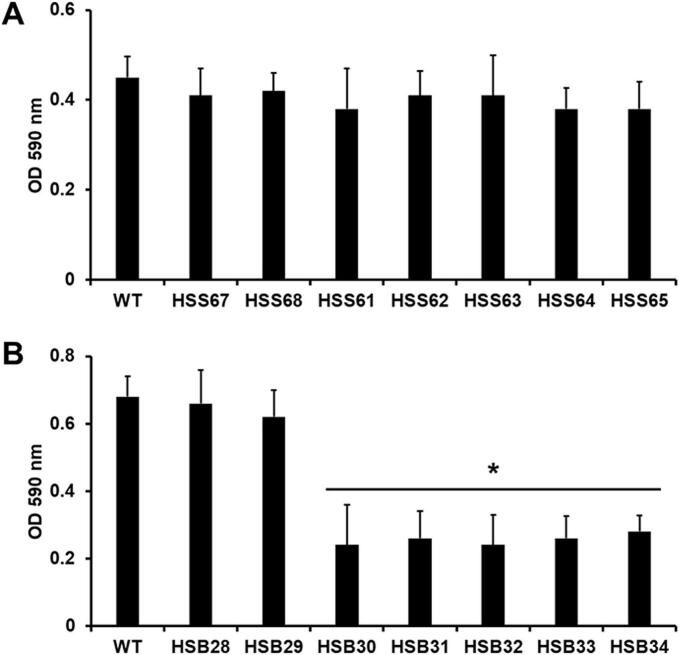
**Biofilm formation in *Sporothrix schenckii* and *Sporothrix brasiliensis PAP1*-silenced strains.** Biofilms were generated in microplates, using yeast-like cells. Non-adherent cells were removed, and biofilms were allowed to mature for 24 h at 37 °C. Biomass was stained with crystal violet and quantified by spectrophotometry. Panel **A** shows data with *S. schenckii* cells, and the WT is 1099–18 ATCC MYA 4821 strain. Panel **B** contains results obtained with *S. brasiliensis* cells, and the WT is the 5110 ATCC MYA 4823 strain. Data are means ± SD of three biological replicates. Results were analyzed with Dunnett's test and then the t-test. *P < 0.05 when compared to WT.

Next, we assessed the ability of yeast-like cells to generate biofilms. For *S. schenckii*, biofilm formation was similar for all the strains analyzed, i.e., the WT, control, and silenced strains (Fig. 4A). For *S. brasiliensis*, the WT and the control strains (HSB28 and HSB29) showed a similar ability to form biofilms (Fig. 4B). However, the *PAP1*-silenced strains (HSB30-HSB34) were poor biolfim producers (Fig. 4B). In terms of extracellular matrix components of biofilms, it was previously reported that glucose, glucosamine, and proteins are the main components of the *Candida parapsilosis* and *Candida tropicalis* biofilms (Al-Fattani and Douglas, 2006; Tóth et al., 2019). When the extracellular matrix components of *Sporothrix* biofilms were analyzed, we found that glucose, glucosamine, and protein levels in the extracellular matrix of biofilms were constant across all the *S. schenckii* strains analyzed (112.8 ± 29.5 μg mL^−1^, 524.6 ± 88.6 μg mL^−1^, and 117.5 ± 21.4 μg mL^−1^, for glucose, glucosamine, and protein, respectively). Similarly, the *S. brasiliensis* biofilm extracellular matrix showed similar levels of the analyzed components across the analyzed strains (136.2 ± 38.5 μg mL^−1^, 598.0 ± 101.1 μg mL^−1^, 148.3 ± 25.6 μg mL^−1^, for glucose, glucosamine, and protein, respectively).

### 3.3 *PAP1* silencing affected *Sporothrix schenckii and Sporothrix brasiliensis* cell wall composition

Since Pap1 was found as one of the major peptidorhamnomannan (PRM) components (García-Carnero et al., 2021), it was likely that the wall may show changes in the composition after *PAP1* gene silencing. The cell wall was acid hydrolyzed to release monosaccharides that compose wall polysaccharides and oligosaccharides, and levels in them were compared among WT and mutant strains (Mora-Montes et al., 2007). WT and mutant strains showed no significant changes in glucose and *N*-acetylglucosamine content (average glucose and *N*-acetylglucosamine cell wall levels in *S. schenckii* WT, control, and silenced strains were 35.8 ± 14.5 and 425.6 ± 28.6 μg monosaccharide mg dry cell wall^−1^, respectively). For *S. brasiliensis* WT, control, and silenced strains, glucose and *N*-acetylglucosamine levels were 42.6 ± 24.5 and 177.6 ± 3 6.5 μg monosaccharide mg dry cell wall^−1^, respectively.

**Fig. 5:**
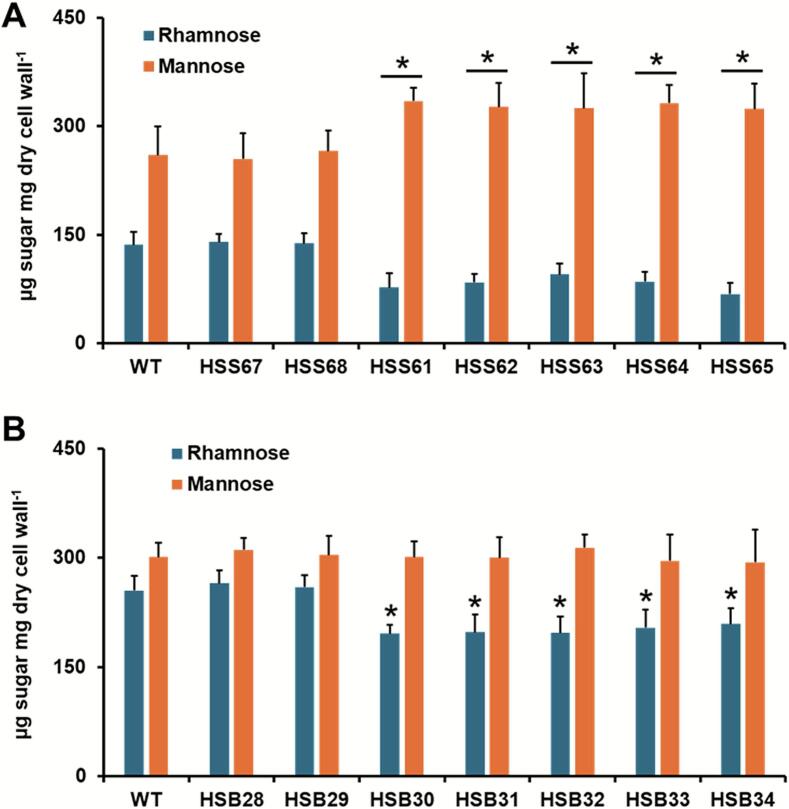
**Cell wall rhamnose and mannose content in *Sporothrix schenckii* and *Sporothrix brasiliensis* wild-type, control, and *PAP1*-silenced mutant strains.** Yeast-like cells were homogenized, cell walls were isolated, cleansed, acid-hydrolyzed, and the resulting monosaccharides were separated and quantified by high-performance anion-exchange chromatography with pulsed amperometric detection. In A, results generated with *S. schenckii* cells. WT, 1099–18 ATCC MYA 4821 strain. In B, results obtained with *S. brasiliensis* strains. WT, 5110 ATCC MYA 4823 strain. For both panels, data are means ± SD of three biological replicates. Results were analyzed with Dunnett's test and then the *t*-test. *P < 0.05 when compared to WT, or control strains (HSS67 and HSS68 in panel A; HSB28 and HSB29 in panel B).

For monosaccharides that are part of glycoproteins, named mannose and rhamnose (Lopes-Bezerra et al., 2018b, Villalobos-Duno et al., 2021), the silenced mutant strains showed differences when compared to the WT or control strains. For *S. schenckii*, the *PAP1*-silenced strains (HSS61-HSS65) showed a significant reduction in the rhamose levels, and this was accompanied by an increment in the cell wall mannose content (Fig. 5A). The control strains HSS67 and HSS68 showed similar rhmanose and mannose levels as the WT strain (Fig. 5A). For the case of *S. brasiliensis*, the *PAP1*-silenced strains, HSB30-HSB34, showed reduced levels of rhamnose but similar mannose levels a those observed in the WT strain. Control strains HSB28 and HSB29 showed similar carbohydrate content as the WT strain (Fig. 5B).

The changes in rhamnose and mannose content suggested changes in the cell wall glycoprotein content. Thus, cell wall *N*-linked glycan, *O*-linked glycan, and protein content were measured in the different strains. We did not observe significant changes in the levels of *N*-linked glycan content in the mutant strains of both species (0.42 ± 0.08 and 0.45 ± 0.06 μg 10^9^ cells ^−1^ on average for *S. schenckii* and *S. brasiliensis* strains, respectively). However, the *O*-linked glycan content showed variations. In the *S. schenckii PAP1*-silenced strains, this was significantly lower and was accompanied by reduced cell wall protein levels (Table 3). The control strains HSS67 and HSS68 showed similar levels of both parameters to the WT strain (Table 3). For *S. brasiliensis*, the *PAP1*-silenced strains showed increased *O*-linked glycan and cell wall content when compared to the WT strain (Table 3). Control strains (HSB28 and HSB29) showed both parameters at similar levels to the WT type strain.

**Table 3:** Cell wall *O*-linked glycan and protein content of *Sporothrix schenckii* and *Sporothrix brasiliensis* wild-type, control, and *PAP1*-silenced strains.

Strain	*O*-linked Glycan (μg 10^9^ cells ^−1^)	Cell Wall Protein Content (μg mg cell wall^−1^)
*S. schenckii*		
1099–18 ATCC MYA 4821	0.48 ± 0.06	178.5 ± 39.4
HSS67	0.46 ± 0.02	182.4 ± 25.4
HSS68	0.42 ± 0.03	180.4 ± 23.2
HSS61	0.32 ± 0.06⁎	125.4 ± 33.8⁎
HSS62	0.35 ± 0.05⁎	120.3 ± 37.4⁎
HSS63	0.32 ± 0.02⁎	112.5 ± 28.4⁎
HSS64	0.29 ± 0.04⁎	118.7 ± 33.0⁎
HSS65	0.35 ± 0.0.4⁎	120.4 ± 38.7⁎
*S. brasiliensis*		
5110 ATCC MYA 4823	0.54 ± 0.07	202.4 ± 36.7
HSB28	0.52 ± 0.03	210.1 ± 42.5
HSB29	0.58 ± 0.0.4	212.6 ± 33.8
HSB30	0.70 ± 0.08⁎	271.5 ± 45.5⁎
HSB31	0.71 ± 0.07⁎	265.4 ± 33.7⁎
HSB32	0.69 ± 0.05⁎	277.6 ± 39.5⁎
HSB33	0.66 ± 0.04⁎	286.8 ± 28.7⁎
HSB34	0.68 ± 0.05⁎	278.3 ± 42.7⁎

⁎ Data are means ± SD of three biological replicates. Results were analyzed with the Dunnett's test and then the t-test. *p* < 0.05 when compared with the values obtained with 1099–18 ATCC MYA 4821 strain (*S. schenckii* strains) or 5110 ATCC MYA 4823 (*S. brasiliensis* strains).

Collectively, these data suggest that *PAP1* silencing led to defects in the cell wall composition in both species.

### 3.4 *PAP1* silencing affected the *Sporothrix schenckii* and *Sporothrix brasiliensis* interaction with human peripheral blood mononuclear cells and monocyte-derived macrophages

**Fig. 6:**
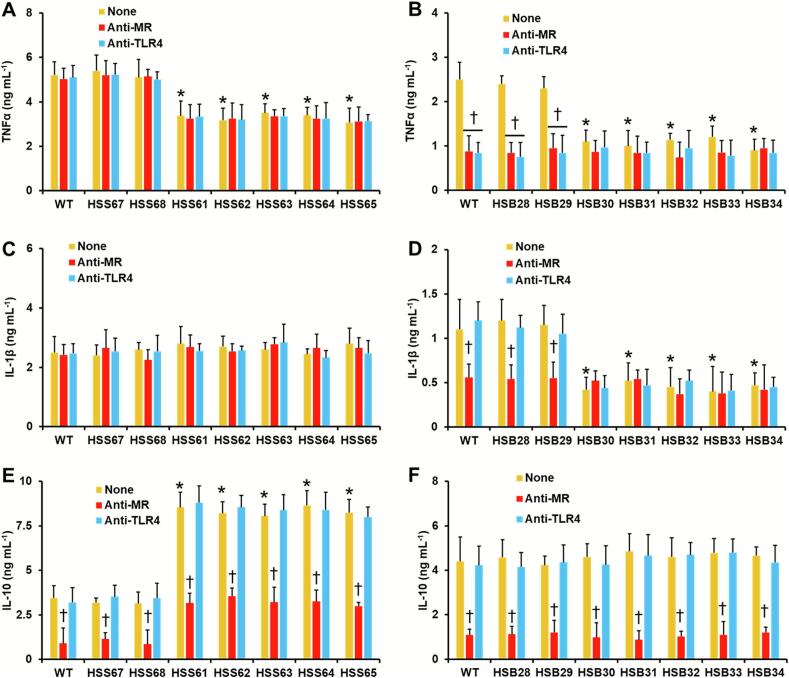
**Cytokine stimulation by human peripheral blood mononuclear cells.** Yeast-like cells and human peripheral blood mononuclear cells were coincubated for 24 h, and the secreted cytokines were quantified by ELISA. Panels **A**, **C**, and **E** are results generated with *S. schenckii* yeast-like cells (WT, 1099–18 ATCC MYA 4821 strain), while panels **B**, **D**, and **F** are results obtained with *S. brasiliensis* strains (WT, 5110 ATCC MYA 4823 strain). None, human cells preincubated with 5 μg mL^−1^ polymyxin B; Anti-MR, human cells preincubated with 5 μg mL^−1^ polymyxin B and 10 μg mL^−1^ anti-mannose receptor antibody. Anti-TLR4, human cells preincubated with 5 μg mL^−1^ polymyxin B and 10 μg mL^−1^ anti-TLR4. Data are means ± SD obtained with samples from eight donors, each assayed in duplicate wells. Results were analyzed with Dunnett's test and then the Mann-Whitney *U* test. *P < 0.05 when compared to strains WT, HSS67, or HSS68 (panels **A**, **C**, and **E**). *P < 0.05 when compared to strains WT, HSB28, or HSB29 (panels **B**, **D**, and **F**). ^†^*P* < 0.05 when compared to the “None” group from the same strain.

Since *PAP1* silencing in both fungal species led to changes in the cell wall composition, we hypothesize that these modifications may affect the interaction with human immune effectors. Thus, we analyzed the ability of the silenced mutants to stimulate cytokine production in human PBMCs. For IL-6, we did not find any significant difference in the stimulation of this cytokine in both the *S. schenckii* and *S. brasiliensis* mutant strains (5.6 ± 0.8 ng mL^−1^ and 1.9 ± 0.4 ng mL^−1^, on average, for *S. schenckii* and *S. brasiliensis* strains, respectively). TNFα stimulation was significantly reduced in cells interacting with the *S. schenckii PAP1*-silenced mutants (HSS61-HSS65) and the *S. brasiliensis PAP1*-silenced mutants (HSB30-HSB34) (Fig. 6A and B). IL-1β stimulation was not affected when the *S. schenckii* mutant cells interacted with human PBMCS, but for *S. brasiliensis PAP1*-silenced mutants, they showed reduced ability to stimulate this cytokine (Fig. 6C and D). The IL-10 stimulation was not affected when the human cells interacted with the *S. brasiliensis PAP1*-silenced strains, but this was significantly higher in cells stimulated with *S. schenckii PAP1*-silenced strains (Fig. 6E and F). The control strains for both species (HSS67, HSS68, HSB28, and HSB29) gave cytokine levels similar to the WT strains (Fig. 6A-6F).

These results may be explained by the absence of the Pap1 peptidic backbone at the cell wall or may be a consequence of the changes with mannose and rhamnose already reported. To discriminate between these possible explanations, the interactions were performed in the presence of anti-MR ot anti-TLR4 antagonistic antibodies, to block the signalling pathways via those membrane receptors. These immune receptors have been previously involved in the sensing of cell wall oligosaccharides (Martínez-Álvarez et al., 2017; García-Carnero et al., 2023). For *S. schenckii* cells, no changes in cytokine levels were observed in cells preincubated with anti-TLR4, or WT, control, and *PAP1*-silenced strains (Fig. 6A, C, and E), indicating no participation of this receptor in the stimulation of these cytokines, as previously reported (García-Carnero et al., 2023). For the case of cells preincubated with anti-MR, only IL-10 production was significantly reduced in all WT, control, and silenced strains (Fig. 6A, C, and E), suggesting that the IL-10 production in cells stimulated with the silenced strains may be due to the increment in cell wall mannose levels. For *S. brasiliensis* cells, TNFα production by WT and control cells was significantly reduced in cells preincubated with either anti-MR or anti-TLR4, suggesting both receptors contribute to the stimulation of this cytokine, as reported (García-Carnero et al., 2023) (Fig. 6B). However, the *PAP1*-silenced strains did not change their ability to stimulate this cytokine when interacting with cells preincubated with anti-MR or anti-TLR4 (Fig. 6B). The IL-1β stimulation by WT and control cells was only sensitive to the presence of anti-MR antibodies, but this effect was not observed in the *PAP1*-silenced strains (Fig. 6D). This same antibody affected the IL-10 stimulation in all the *S. brasiliensis* strains tested, i.e., WT, control, and silenced strains (Fig. 6F). Control experiments in which immune cells were preincubated with an irrelenvant antibody gave similar cytokine levels as the WT strains (data not shown).

**Fig. 7:**
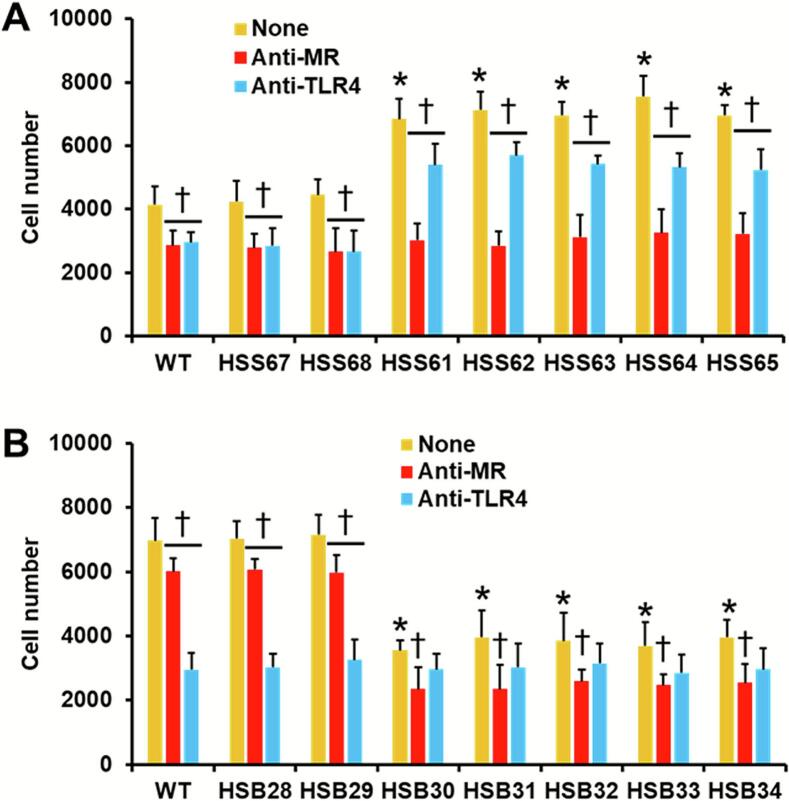
**Phagocytosis of *Sporothrix schenckii* and *Sporothrix brasiliensis PAP1*-silenced strains.** Yeast-like cells were labeled with Acridine Orange and used to interact with human monocyte-derived macrophages for 2 h at 37 °C and 5 % (v/v) CO_2_. Then, macrophages were collected and analyzed by flow cytometry. Macrophages that were interacting with at least one red fluorescent yeast-like cell were included in the analysis. None, human cells preincubated with 5 μg mL-1 polymyxin B. Anti-MR, human cells preincubated with 5 μg mL^−1^ polymyxin B and 10 μg mL^−1^ anti-mannose receptor antibody. Anti-TLR4, human cells preincubated with 5 μg mL^−1^ polymyxin B and 10 μg mL^−1^ anti-TLR4. Panel **A**, results generated with *S. schenckii* yeast-like cells (WT, 1099–18 ATCC MYA 4821 strain); while in panel **B** are results obtained with *S. brasiliensis* strains (WT, 5110 ATCC MYA 4823 strain). Data are means ± SD obtained with samples from eight donors, each assayed in duplicate wells. Results were analyzed with Dunnett's test and then the Mann-Whitney U test. *P < 0.05 when compared to strains WT, HSS67, or HSS68 (panel **A**). *P < 0.05 when compared to strains WT, HSB28, or HSB29 (panel **B**). ^†^*P* < 0.05 when compared to the “None” group from the same strain. (For interpretation of the references to colour in this figure legend, the reader is referred to the web version of this article.)

Next, we analyzed the phagocytic interaction with human monocyte-derived macrophages. We focused our analysis only on the cells interacting with yeast-like cells that were within mature phagolysosomes, since previous experiments have already demonstrated that most of the cells are in this stage after the incubation time (Gómez-Gaviria et al. 2023b). Human cells showed similar ability to phagocyte WT and control cells, an observation for both fungal species (Fig. 7A and B). However, the *PAP1* silencing had an opposite effect in both species. In *S. schenckii*, the monocyte-derived macrophages showed increased ability to phagocytose the silenced mutants, while phagocytosis was reduced when interacting with the *S. brasiliensis PAP1*-silenced mutans (Fig. 7A and B). When the contribution of MR and TRL4 in the phagocytic process was assessed, the WT and control cells of both fungal species were significantly less phagocytosed than untreated macrophages (Fig. 7A and B). The phagocytosis of *S. schenckii PAP1*-silenced strains was significantly reduced when immune cells were preincubated with either anti-MR or anti-TLR4 antibody, but in *S. brasiliensis*, only anti-MR antibody significantly reduced the phagocytosis of *PAP1*-silenced strains (Fig. 7A and B). Incubations with an isotype-matched irrelevant antibody showed phagocytosis levels as untreated monocyte-derived macrophages (data not shown).

**Fig. 8:**
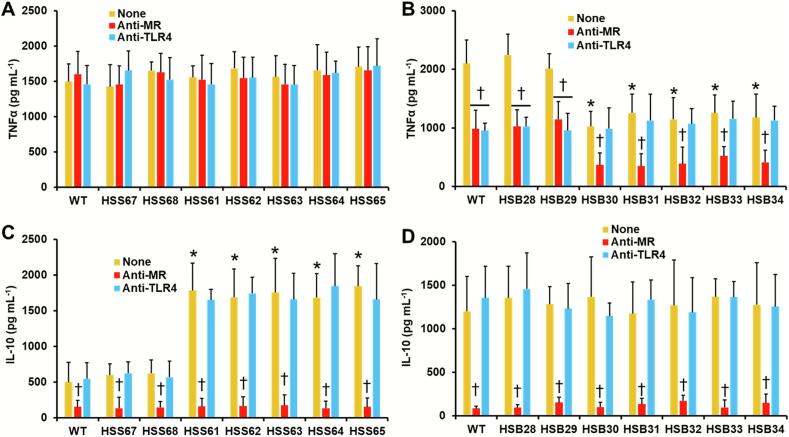
**Cytokine stimulation by human monocyte-derived macrophages.** Yeast-like cells and monocyte-derived macrophages were coincubated for 24 h, and the secreted cytokines were quantified by ELISA. Panels **A** and **C** are results generated with *S. schenckii* yeast-like cells (WT, 1099–18 ATCC MYA 4821 strain); while panels **B** and **D** are results obtained with *S. brasiliensis* strains (WT, 5110 ATCC MYA 4823 strain). None, human cells preincubated with 5 μg mL^−1^ polymyxin B. Anti-MR, human cells preincubated with 5 μg mL^−1^ polymyxin B and 10 μg mL^−1^ anti-mannose receptor antibody. Anti-TLR4, human cells preincubated with 5 μg mL^−1^ polymyxin B and 10 μg mL^−1^ anti-TLR4. Data are means ± SD obtained with samples from eight donors, each assayed in duplicate wells. Results were analyzed with Dunnett's test and then the Mann-Whitney U test. *P < 0.05 when compared to strains WT, HSS67, or HSS68 (panels **A** and **C**). *P < 0.05 when compared to strains WT, HSB28, or HSB29 (panels **B** and **D**). cells. ^†^*P* < 0.05 when compared to the “None” group from the same strain.

We also measured cytokine production upon stimulation with yeast-like cells. For cells stimulated with *S. schenckii*, there were no differences in TNFα production when WT. control and *PAP1*-silenced mutants were compared (Fig. 8A). However, IL-10 production was significantly increased in cells stimulated with the *PAP1*-silenced strains (HSS61-HSS65). WT and control strains stimulated similar IL-10 levels (Fig. 8C). For *S. brasiliensis*, TNFα stimulation was reduced when human cells interacted with the *PAP1*-silenced strains (Fig. 8B). IL-10 production was similar for WT, control, and silenced strains (Fig. 8D). When the contribution of MR and TLR4 was analyzed, preincubation of monocyte-derived macrophages with either anti-MR or anti-TLR4 did not affect TNFα production (Fig. 8A). For IL-10, a significant reduction was observed only when cells were preincubated with anti-MR, and this was at the same level in all the analyzed strains (Fig. 8C). For *S. brasiliensis*, preincubation with either anti-MR or anti-TLR4 significantly reduced the TNFα levels stimulated by WT or control cells (Fig. 8B). For the *PAP1*-silenced strains, only preincubation with anti-MR reduced TNFα stimulation (Fig. 8 B). Concerning IL-10 stimulation, this was significantly reduced only when macrophages were preincubated with anti-MR (Fig. 8D). Control interactions where immune cells were preincubated with an irrelevant isotype-matched antibody did not change the cytokine production, when compared to untreated cells (data not shown). Collectively, these results indicate that *PAP1* silencing affected the interaction of both *S. schenckii* and *S. brasiliensis* with human PBMCs and monocyte-derived macrophages.

### 3.5 The *Sporothrix schenckii* and *Sporothrix brasiliensis Pap1*-silenced mutant showed virulence attenuation

**Fig. 9:**
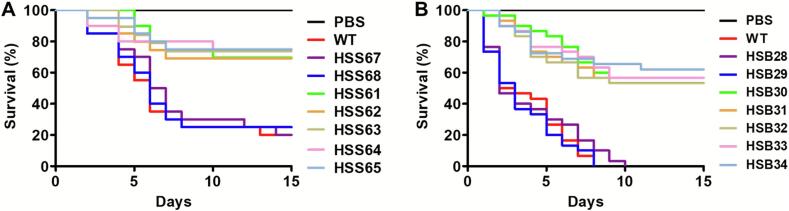
**Killing curves of *Galleria mellonella* larvae inoculated with *Sporothrix schenckii* and *Sporothrix brasiliensis* WT, control, and *PAP1*-silenced strains.** The *G. mellonella* larvae were inoculated with 10 μL of a yeast-like cell suspension at 1 × 10^7^ cells mL^-1,^ and survival was monitored daily for two weeks. Mortality is shown in Kaplan–Meier plots. In A, data generated with *S. scehcnkii* strains; while in B are shown the results with *S. brasiliensis* strains. Data were analyzed with the Log-rank test. In both panels, curves generated with the five *PAP1*-silenced strains were not significantly different among themselves but were significantly different from the curves generated with the WT or control strains (*P* < 0.05). In A, WT, 1099–18 ATCC MYA 4821 strain. Control strains are HSS67 and HSS68. In B, WT, 5110 ATCC MYA 4823 strain. Control strains are HSB28 and HSB29. For each strain, a total of 30 larvae were included in the study. In both panels, PBS, a larval group inoculated only with phosphate saline buffer.

The contribution of *PAP1* to *Sporothrix* virulence was assessed in the alternative host *G. mellonella* larva, where an experimental model of disseminated sporotrichosis was previously established (Clavijo-Giraldo et al., 2016). Larvae infected with *S. schenckii* WT and control strains (HSS67 and HSS68) showed a median survival of 6.25 ± 1.3 days with an accumulated mortality at the end of the observation period of 77.5 ± 8.6 % (Fig. 9A). When the *PAP1*-silenced strains were used to inoculate larvae, the five experimental groups showed similar killing curves (Fig. 9A). The mean survival was higher than 15 days for the five groups, and the mortality rate on average was 27.5 ± 5.5 %. In the case of larvae infected with *S. brasiliensis* WT or control strains (HSB28 and HSB29), the median survival was 2.8 ± 0.9 days, with a mortality rate of 100 % for the three strains (Fig. 9B). All animal groups infected with the *S. brasiliensis PAP1*-silenced strains showed a median survival of more than 15 days, and the mortality rate was on average 41.0 ± 5.0 % (Fig. 9B).

We also quantified the fungal burden from larvae infected with the different strains. For both species, WT, control, and *PAP1*-silenced strains showed similar fungal loads, indicating that all the strains had the same ability to adapt to the host milieu (Table 4).

**Table 4:** Fungal load, hemocyte levels, phenol oxidase activity, and cytotoxicity in *Galleria mellonella* larvae infected with *Sporothrix schenckii* and *Sporothrix brasiliensis* wild-type, control, and *PAP1*-silenced strains.

Strain	Colony forming units (x 10^5^)	Hemocyte levels (×10^6^ mL^−1^)	Phenol oxidase^a^	Cytotoxicity (%)^b^
PBS^c^	0.0 ± 0	3.2 ± 0.5	0.6 ± 0.4	10.5 ± 4.7
*S. schenckii*				
1099–18 ATCC MYA 4821	2.7 ± 0.8	8.3 ± 0.6	3.1 ± 0.5	78.2 ± 12.4
HSS67	3.1 ± 0.8	7.9 ± 0.9	2.8 ± 0.6	74.6 ± 10.5
HSS68	2.9 ± 0.6	8.1 ± 0.8	3.3 ± 0.5	79.0 ± 13.8
HSS61	2.9 ± 0.8	3.9 ± 0.3*	0.9 ± 0.8*	48.7 ± 11.5*
HSS62	3.2 ± 0.6	3.5 ± 0.6*	0.8 ± 0.7*	44.8 ± 9.8*
HSS63	3.3 ± 0.4	3.2 ± 0.8*	1.3 ± 0.6*	46.0 ± 14.5*
HSS64	2.6 ± 0.6	4.0 ± 0.5*	0.9 ± 0.4*	49.8 ± 8.8*
HSS65	2.8 ± 0.7	3.6 ± 0.7*	1.1 ± 0.4*	42.8 ± 10.1*
*S. brasiliensis*				
5110 ATCC MYA 4823	2.9 ± 0.9	9.4 ± 0.8	4.2 ± 0.7	92.5 ± 7.7
HSB28	2.8 ± 0.6	9.6 ± 1.1	4.4 ± 0.5	91.5 ± 6.8
HSB29	3.3 ± 0.6	9.8 ± 0.9	3.8 ± 0.9	86.2 ± 9.7
HSB30	3.0 ± 0.9	4.4 ± 0.6*	1.9 ± 0.5*	32.4 ± 16.4*
HSB31	2.8 ± 0.5	4.9 ± 0.7*	2.4 ± 0.6*	33.5 ± 14.8*
HSB32	2.6 ± 0.8	4.6 ± 0.5*	2.2 ± 0.9*	40.5 ± 11.6*
HSB33	2.6 ± 0.6	4.8 ± 1.0*	1.8 ± 0.8*	38.9 ± 21.5*
HSB34	3.2 ± 0.8	5.0 ± 0.9*	1.7 ± 0.9*	36.1 ± 18.6*

^a^ Defined as the Δ_490nm_ per min per μg protein. ^b^Cell-free lactate dehydrogenase was quantified as a measure of cell cytotoxicity. Data were normalized to those obtained with lised hemocytes, which represent 100%. ^C^ Larva inoculated only with phosphate saline buffer. *p < 0.05 when compared with WT or control strains. For *S. schenckii*, WT is 1099-18 ATCC MYA 4821, and control strains are HSS67 and HSS68. For *S. brasiliensis*, WT is 110 ATCC MYA 4823, and control strains are HSB28 and HSB29. Data are from groups containing 10 larvae analyzed in duplicate.

Hemocyte levels and phenol oxidase activity in hemolymph are two parameters useful to assess the immunological fitness when *G. mellonella* larvae are infected with a pathogen (Pereira et al., 2018). Both parameters increased when larvae were infected with WT or control strains in both species, suggesting an immunological response by the host (Table 4). However, both parameters were significantly reduced in the animal groups challenged with the *PAP1*-silenced strains from both *Sporothrix* species (Table 4). Cell cytotoxicity was significantly high in the hemolymph of larvae inoculated with WT or control strains from both species, but this was reduced in the hemolymph of larvae inoculated with *PAP1*-silenced mutants from both *S. schenckii* and *S. brasiliensis* (Table 4). Collectively, these data suggest that *PAP1* silencing affects the *S. schenckii* and *S. brasiliensis* virulence.

## 4 Discussion

To our knowledge, this is the first study describing the back-to-back phenotypical characterization of mutant strains in a particular gene in both *S. schenckii* and *S. brasiliensis*. In addition, this study adds valuable data to the scarce information on the molecular level of virulence factors in *Sporothrix* spp. (García-Carnero and Martínez-Álvarez, 2022). The fact that high silencing levels could be achieved and the normal cell phenotype and doubling times in both species suggest that *PAP1* is not an essential gene for fungal growth or dimorphism. It has been previously suggested that Pap1 is not a moonlighting protein. Instead, it is predicted to be a highly intrinsically disordered protein with no obvious domain motifs for canonical intracellular function. Currently, the sole cellular function reported is as an adhesin (García-Carnero et al., 2021). These results support this observation. The fact that *PAP1* is highly expressed in yeast-like cells and cells interacting with ECM, human cells, or within *G. mellonella* suggests that this protein is involved in the interaction with the host.

Despite the high similarity percentage between Pap1 from *S. schenckii* and *S. brasiliensis*, the increased length in the *S. brasiliensis* polypeptide made it no obvious to predict that the protein may have adhesive properties, similar to those described in *S. schenckii* Pap1. Even though we do not have a detailed structural analysis of the proteins, one hypothesis that emerges from our observations is that the additional amino acids found in the *S. brasiliensis* Pap1 may hinder interaction with elastin and favor binding to thrombospondin 1. It has been previously reported that HeLa cells produce thrombospondin-1 (Kang et al., 2007), and this may be behind the observation that *S. brasiliensis* bound to HeLa cells more avidly than *S. schenckii* cells.

Adhesion, germination and growth, maturation, and dispersion are the main stages of fungal biofilm formation (Wang et al., 2024). Our results suggested that Pap1 has no role in *S. schenckii* biofilm formation, but it is required for proper biofilm formation in *S. brasiliensis*. These cells did not show defects in growth rate and in the extracellular matrix, suggesting that the reduced biofilm formation in this species may be because few cells could adhere to the plastic surface, i.e., there was a defect in the adhesion stage. Moreover, likely, *S. brasiliensis* Pap1 is also relevant for cell-cell interactions within the biofilm.

In terms of cell wall changes in the *PAP1*-silenced mutants, the results suggested that Pap1 is an abundant protein in at least the *S. schenckii* cell wall, as its silencing significantly reduced cell wall protein content. This result is similar to that generated in *GP70*-silenced mutants (López-Ramírez et al., 2024), indicating that both proteins Pap1 and Gp70 are two of the main cell wall proteins in this species, stressing the relevance of cell adhesion for *S. schenckii* yeast-like cells' pathogenesis. The fact that cell wall protein content increased in the *S. brasiliensis PAP1*-silenced strains suggests a compensatory mechanism not observed in *S. schenckii* and that it is also an abundant protein whose absence is compensated by others. It is worth noting that this compensatory mechanism was not activated in *S. brasiliensis GP70*-silenced strains (Padró-Villegas et al., 2025), suggesting Pap1 plays a more significant role than Gp70 in this species. This is in line with previous observations, where low cell wall Gp70 levels were associated with higher virulence in *S. brasiliensis* (Castro et al., 2013). The changes in rhamnose are in line with reduced levels of *O*-linked glycans within the PRM complex, as the main *O*-linked glycan found in it contains rhamnose-conjugated moieties (Lopoes-Bezerra, 2011). This hypothesis may explain the changes observed in *S. schenckii*, but in the case of *S. brasiliensis*, the increment in *O*-linked glycan content is in line with the proposed compensatory mechanism activated once Pap1 levels are reduced. Mannose increment in the *S. schenckii* silenced mutants but not in *S. brasiliensis* mutants suggests an additional compensatory mechanism in the former. Whether this positively affects protein glycans or glycolipids remains to be solved.

In terms of interaction with effectors of the innate immunity, named human PBMCs and monocyte-derived macrophages, the results suggested that the changes in cytokine production and phagocytosis were a consequence of the modifications in the cell wall composition, in particular in rhamnose and mannose content. A current limitation of our study is that we cannot formally discard that the peptidic backbone itself of Pap1 may be associated with the changes in the interaction with immune cells. It has been demonstrated that a protein may be recognized as a pathogen-associated molecular pattern on the surface of some pathogenic agents. Examples of these are RcCDI1 from *Rhynchosporium commune*, which is recognized by *Nicotiana benthamiana* NbBAK1 (Franco-Orozco et al., 2017), and *Klebsiella pneumoniae* KpOmpA and flagellin that are sensed via TLR2 and TLR5, respectively, in human NK cells (Chalifour et al., 2004). Nevertheless, our results suggest an important role of Pap1 during immune sensing.

The fact that the human PBMCS did not show further cytokine changes when preincubated with anti-MR or anti-TLR4 and challenged with *PAP1*-silenced strains suggests that these two immune receptors are relevant and key players during the interaction with *Sporithrix* cells. In line with this observation, previous results have indicated that TLR4 is a relevant player in the mouse model of experimental sporotrichosis (Carlos et al., 2009; Sassá et al., 2012; Rossato et al., 2019). Currently, TLR4 is thought to recognize cell wall rhamnoconjugates (Tamez-Castrellón et al., 2021), placing rhamnose and mannose immune sensing as relevant as the β-1,3-gulcan sensing in both *S. schenckii* and *S. brasiliensis* (Jellmayer et al., 2017; Yoshikawa et al., 2025).

The results indicated that the increment in cell wall mannose in the *S. schenckii PAP1*-silenced strains was behind the increased phagocytosis by human monocyte-derived macrophages, as the anti-MR antibody inhibited the fungal uptake to levels comparable to the WT control strain. Currently, dectin-1 has been placed as the main player in *Sporothrix* uptake by human and murine macrophages (Jellmayer et al., 2017, Gómez-Gaviria, Martínez-Duncker, García-Carnero and Mora-Montes, 2023b), but results generated here indicate that MR is also a relevant player in *S. schenckii* phagocytosis by macrophages. Similar observations have been reported during *C. albicans* and *Cryptococcus neoformans* uptake (Syme et al., 2002; Heinsbroek et al., 2008). Results generated with *S. brasiliensis* cells underscore the relevance of rhamnoconjugates not only for cytokine production but also for phagocytosis of these fungal species. This species-specific difference may be linked to the cell wall rhamnose levels in both *S. schenckii* and *S. brasiliensis*, being the latter with the highest cell wall rhamnose content (Lopes-Bezerra et al., 2018b, Villalobos-Duno et al., 2021).

The Pap1 role in virulence was previously suggested in *S. schenckii*, where anti-Pap1 antibodies were used to preincubate yeast-like cells, and these showed reduced ability to kill *G. mellonella* larvae (García-Carnero et al., 2021). Results generated here confirmed this observation and expanded our current knowledge on *PAP1* in *S. brasiliensis*, where it is also relevant for fungal virulence. The lack of changes in colony-forming units collected from hemolymph makes it unlikely these results may be explained by defects in the ability of the silenced mutant to adapt to the host milieu. In addition, the changes in cytotoxicity and in the measured immunological parameters support the idea that Pap1 is relevant for *S. schenckii* and *S. brasiliensis* virulence. One interesting observation is that silenced mutants in Gp70, a cell wall protein with adhesive properties to fibronectin and laminin in both *S. schenckii* and *S. brasiliensis* (López-Ramírez et al., 2024; Padró-Villegas et al., 2025), also showed virulence attenuation. Even though Gp70 is not part of the PRM, it is feasible to conceive that both proteins, Gp70 and Pap1, may work together during the interaction with ECM components, and therefore, may have a collaborative effect on *Sporothrix* virulence. This hypothesis remains to be addressed.

In conclusion, we showed evidence indicating that *S. schenckii* and *S. brasiliensis PAP1* code for a protein with adhesive properties to ECM components and epithelial cells. The latter also participates in the establishment of biofilms. This is an abundant cell wall protein that contributes to *Sporothrix* virulence and the interaction with cells from innate immunity.

## CRediT authorship contribution statement

**Leonardo Padró-Villegas:** Writing – review & editing, Writing – original draft, Visualization, Methodology, Investigation, Formal analysis, Conceptualization. **Julieta I. Aguilera-Domínguez:** Writing – original draft, Visualization, Investigation, Formal analysis, Conceptualization. **Luz A. López-Ramírez:** Writing – original draft, Supervision, Project administration, Methodology, Investigation, Formal analysis, Conceptualization. **Manuela Gómez-Gaviria:** Writing – review & editing, Writing – original draft, Visualization, Validation, Methodology, Investigation, Formal analysis, Data curation. **Iván Martínez-Duncker:** Writing – review & editing, Writing – original draft, Validation, Supervision, Resources, Methodology, Investigation, Funding acquisition, Formal analysis, Data curation, Conceptualization. **Laura C. García-Carnero:** Writing – original draft, Visualization, Validation, Methodology, Investigation, Formal analysis, Conceptualization. **Joaquín O. Chávez-Santiago:** Writing – review & editing, Visualization, Validation, Software, Methodology, Investigation, Formal analysis. **Patricia Ponce-Noyola:** Writing – review & editing, Writing – original draft, Validation, Supervision, Methodology, Investigation, Funding acquisition, Formal analysis, Conceptualization. **Héctor M. Mora-Montes:** Writing – review & editing, Writing – original draft, Visualization, Validation, Supervision, Resources, Project administration, Methodology, Investigation, Funding acquisition, Formal analysis, Data curation, Conceptualization.

## Declaration of competing interest

The authors declare that they have no known competing financial interests or personal relationships that could have appeared to influence the work reported in this paper.

## Data Availability

Data will be made available on request.
